# Behavioural risk factors and healthy life expectancy: evidence from two longitudinal studies of ageing in England and the US

**DOI:** 10.1038/s41598-020-63843-6

**Published:** 2020-04-24

**Authors:** Paola Zaninotto, Jenny Head, Andrew Steptoe

**Affiliations:** 1 Department of Epidemiology and Public Health, University College London, London, UK; 2 Department of Behavioural Science and Health, University College London, London, UK

**Keywords:** Diseases, Risk factors

## Abstract

We examined whether the co-occurrence of four behavioural risk factors (alcohol consumption, smoking, physical inactivity and obesity) is associated with disability-free and chronic disease-free life expectancy similarly in two longitudinal studies of ageing in England and the United States. Data were from 17,351 individuals aged 50+ from the US Health and Retirement Study (HRS) and, 10,388 from the English Longitudinal Study of Ageing (ELSA), from 2002 to 2013. Disability-free life expectancy was estimated using repeat measures of limitations with instrumental activities and activities of daily living and, chronic disease-free life expectancy was based on chronic health conditions. Multistate life table models were used to estimate sex-specific health expectancy at the ages of 50, 60 and 70. In both countries and at all ages, there was a clear gradient towards shorter health expectancy with increasing number of behavioural risk factors. Compared to people with 2+ behavioural risk factors, in both countries, those with no behavioural risk factors could expect to live up to 11 years longer without disability and, up to 12 years longer without chronic conditions. Individual and co-occurring behavioural risk factors were strongly associated with shorter healthy life expectancy in both countries, attesting to the robustness of the contribution of lifestyle factors on health expectancy.

## Introduction

Over the last century life expectancy has increased dramatically in both the United States (USA) and United Kingdom (UK). However, there are concerns that gains in healthy life expectancy are not keeping pace with those in life expectancy. In the USA^1^, England^2^ and globally^3^, a large proportion of older adults experience high levels of disability and chronic conditions which, in turn, pose financial challenges for governments and health-care systems worldwide.

Life expectancy has been widely used as an indicator of health, but in recent years there has been an increasing interest in the quantification of the quality of remaining years of life^3^. Health expectancy combines data on both mortality and morbidity or disability^4^, as such it gives an estimate of the remaining number of years of life expected to live in favorable states of health or without disability. Health expectancy indicators have been widely used to compare the health in different populations, to monitor time trends and, to explore health inequalities in population.

Self-rated health has often been used to compute “healthy life expectancy”, whereas “disability-free life expectancy”, is commonly computed using limitations with activities of daily living (ADL) and instrumental activities of daily living (IADL)^4,5^. Chronic conditions have been used to compute “chronic disease-free or morbidity-free life expectancy”. Self-rated health is based on subjective health status, therefore, due to cultural differences, identical questions may not mean the same to people in different countries. Disability-free life expectancy is one of the most widely accepted measure of health expectancy^5^; it has been recommended in cross-country comparative studies^4^ because it is less sensitive to cultural factors. Although chronic disease-free life expectancy is less widely used^5^, compared to healthy and disability-free life expectancy, it is more relevant at older ages since an increasing number of older people report chronic conditions^6^.

Increasing the number of years that older people live in optimal health is considered a major priority^7^. As a consequence, in recent years a body of research has emerged on the potential determinants of health expectancy, with a focus on modifiable risk factors, such as alcohol consumption, smoking, physical inactivity and obesity^8–15^. These studies have reported that these factors, both individually^8–10,13–15^ and jointly^10–15^, are associated with healthy life expectancy. However, existing studies have a series of shortcomings which hamper the interpretation of results. These weaknesses include: a focus on special (non-representative) populations^8,10,13,15^, so reducing the generalizability of results; a short duration of follow-up^11^ and a study population aged <75^8,9,11–15^, hampering understanding of the natural history of the ageing process across the older age life course. Lastly, given the importance of chronic conditions in older adults, it is surprising that only a few of these studies have computed a measure of health expectancy based on chronic conditions^8–10,12,14,15^.

Accordingly, the aim of this study was to examine the extent to which the co-occurrence of alcohol consumption, smoking, physical inactivity and obesity predicted total life expectancy as well as disability-free life expectancy and chronic disease-free life expectancy, in two nationally representative longitudinal studies of older people, aged 50 and over, in England and the United States with a follow-up of 10+ years. We also investigated whether, these behavioural risk factors, showed different associations with health expectancy in the two countries, because of differences in the cultural embedding of these lifestyle factors. For example, self-perceptions of overweight have declined markedly among USA adults over the past 20 years^16^, while older people in the USA show larger discrepancies between self-rated and objective physical activity compared with their English counterparts^17^. These comparisons were facilitated by the use of harmonized data from the Gateway to Global Aging Data.

## Methods

Detailed information about data harmonization and statistical methods is available in the Supplementary information.

### Data

We used data from two prospective cohort studies of ageing: the English Longitudinal Study of Ageing (ELSA) in England and the Health and Retirement Study (HRS) in the USA. Established 10 years after HRS, ELSA was designed to be comparable in terms of population sampling, periodicity, and content (including the specific wording of questions)^18,19^. The two studies have been described in detail elsewhere^20,21^. To maximize comparability between the two studies, we used harmonized data files, from 2002/2003 to 2012/2013, available from The Gateway to Global Aging Data (g2aging.org), which is a data and information platform developed to facilitate cross-country analyses. The data files provided a set of harmonized or identically defined variables. In both cohorts, we included people aged 50+ with valid data on health, behavioural risk factors and wealth, resulting in analytical samples of 10,338 (out of the 11,391 ELSA members in 2002/2003) and 17,351 (out of the 17,758 HRS members in 2002 aged 50 and over) (refreshment samples added after 2002 are excluded from these analyses).

The sample size at subsequent waves was: 7,874 in ELSA and 15,435 in HRS; 6,634 in ELSA and 13, 919 in HRS; 5,736 in ELSA and 12,720 in HRS; 5,291 in ELSA and 11,262 in HRS; 4, 695 in ELSA and 5,528 in HRS.

### Outcome measures

We measured health expectancy using two health indicators: the presence of disability and having a chronic health condition.

#### Disability

At each wave, in both studies, all participants were asked whether they had difficulties in performing activities of daily living (ADL) (e.g., dressing, walk across a room, bathing or showering, eating, getting in/out of bed, using the toilet) and instrumental activities of daily living (IADL) (e.g., using a map, preparing a hot meal, shopping for groceries, making phone calls, taking medications, managing money)^22^. Responses were summed and categorized as no disability (0 or 1 ADL/IADL) and disability (2+ ADL/IADL). The cutoff of 2 or more ADL or IADL was chosen based on the average number of ADL or IADL limitations reported by people (in ELSA) who at baseline were in receipt of health or disability benefits^22^. Health expectancy based on disability is named here as disability-free life expectancy.

#### Chronic conditions

At each wave of the study respondents were asked ‘has a doctor ever told you that you have …’: (1) coronary heart disease (2) stroke, (3) lung disease (chronic bronchitis or emphysema), (4) cancer, (5) diabetes and (6) arthritis. Chronic disease-free life expectancy was defined as having one or more of these conditions. The presence of chronic conditions at baseline (first observation included in analysis) includes any chronic conditions reported before the age of 50 from available information on respondents.

*Mortality* up to March 2013 was ascertained from linked register data for ELSA and through linkages to the National Death Index and reports from survivors for HRS^22^.

### Behavioural risk factors

Body Mass Index (BMI) was computed using self-reported body weight and height in HRS; in ELSA study nurses measured weight and height in the participants’ homes at alternative waves. Obesity was defined as BMI ≥ 30Kg/m^2^. Smoking status was categorized into “Never or former smoker” and “Current smoker”. Frequency of alcohol consumption was dichotomized into “Less than 5 days a week” “5–7 days a week”. Physical activity was defined as being “physically active” if taking part in vigorous physical activity for 2+ days a week (3+ in HRS) and “physically inactive” otherwise. Co-occurrence of the four behavioural risk factors (alcohol, smoking, physical inactivity and obesity) was computed as the sum of the risk factors and recoded as 0, 1 or 2 and more.

### Socioeconomic status

The socioeconomic status indicator used in the analyses was total household wealth^23^ defined as the sum of net financial wealth and net housing wealth less all debts. The continuous variable was divided into three groups (i.e. each containing 33% of the sample).

### Statistical analyses

Total length of time in study was 10 years (from 2002/2003 to 2012/2013, average follow-up 6 years). By the end of follow-up 5,528 deaths occurred in the USA sample and 2,208 in the English sample.

A full description of the methodology used to compute healthy life expectancies is available in Supplementary information. Briefly, we used multistate life table models^4,22,24^ suitable for longitudinal data to estimate total life expectancy, disability-free and chronic disease-free life expectancy, from the ages of 50 to 100. We defined the following three health states: healthy, unhealthy and dead. For disability-free life expectancy, there were four possible transitions between the health states, namely: healthy to unhealthy (onset), unhealthy to healthy (recovery), healthy to dead, unhealthy to dead. For chronic disease-free life expectancy, there were only three possible transitions as, by definition, recovery was not possible.

We used the Stochastic Population Analysis for Complex Events (SPACE) program^24^ in SAS 9.2 to estimate multistate life table functions. There are two main components to this program: the data component, which prepares the input datasets, and the statistical component, in which transition probabilities and the multistate life table functions and their variances are estimated. Specifically, during the statistical component age-specific transition probabilities for all possible transitions are estimated from the data using multinomial logistic regression with age, sex, wealth tertiles, behavioural risk factors, and the interaction term between age and behavioural risk factors. Health expectancies for ages 50+ are then calculated based on these estimated transition probabilities using a stochastic (micro-simulation) approach. By using micro-simulation it is possible to simulate the life paths of the members of the population in order to derive several summary statistics of the population dynamics. For each study separately, the program generated individual trajectories for a simulated cohort of 100,000 persons with distributions of covariates at the starting point based on the observed study-specific prevalence by five year age group and sex. Analyses were run for combined behavioural risk factors and for each of the four behavioural risk factors separately. Variability for these multistate life table estimates (variances, standard errors and corresponding 95% confidence intervals) were computed using a bootstrap method with 500 replicates for the whole analysis process (multinomial analysis and simulation steps). The method deals with unevenly spaced observations^4,22,24^.

## Results

Baseline (2002) characteristics of the study cohorts are presented in Table 1. The prevalence of disability was around 11% in men in England and as high as 23% in women in USA. Chronic health conditions were most common among men (75%) and women (78%) in USA. In England 18% of men and 15% of women did not have any of the four behavioural risk factors, in the USA the corresponding figures were 27% in men and 22% in women (p < 0.001). Physical inactivity was the most common behavioural risk factor in England and the USA.

**Table 1:** Baseline sample characteristics, by gender and cohort study, England and Unites States 2002.

	England (ELSA)		USA (HRS)	
	Men (n = 4,718)	Women (n = 5,620)	Men (n = 7,308)	Women (n = 10,043)
Age Mean (S.D.)	64.5 (9.7)	65.0 (10.2)	68.8 (9.1)	69.0 (10.5)
**Wealth tertiles %**				
High	35.2	31.4	38.7	37.7
Middle	33.6	34	33	32.6
Low	31.2	34.6	28.3	29.7
Disability %	11.1	14.4	14.5	22.7
Chronic health conditions %*	44.8	50.3	74.6	78.3
**Co-occurrence of behavioural risk factors %**				
0	17.5	14.6	26.5	22.0
1	41.2	44.0	44.7	49.5
≥2	41.3	41.4	28.8	28.5
Smoking, %	18.1	18.0	12.8	10.6
Physical inactivity, %	57.2	67.3	55.7	67.2
High frequency of alcohol consumption %	35.3	21.6	14.1	6.1
Obesity, %	23.5	27.9	24.6	25.9

Percentages and mean are estimated using sampling weights. * Heart disease, stroke, chronic lung disease, cancer, arthritis, or diabetes.

In Supplementary Table 1, we report the prevalence of single chronic conditions at baseline by gender and study. Older Americans reported higher prevalence of each condition, and in particular, a substantially higher prevalence of arthritis.

### Co-occurence of health behavioural risk factors and total life expectancy

In Table 2 we report total life expectancy at the ages of 50, 60 and 70, by gender and country, according to the number of behavioural risk factors. At age 50, the average number of years that people with no behavioural risk factors can expect to live was 36 for men and 40 for women in England and, 35 in men and 39 in women in USA. Total life expectancy at age 50 (sum of healthy and unhealthy remaining years of life) was 8 years shorter in men and women in England and the USA engaging in two or more behavioural related risk factors, compared to those with no behavioural risk factors. Total life expectancy at the age of 60 for men with no behavioural risk factors was 27 and 26 years in England and the USA respectively (30 and 29 years among women), compared to 20 years in men reporting two or more behavioural risk factors (24 and 23 years in women from England and the USA respectively). At the age of 70, the difference in total life expectancy between people without behavioural risk factors and those reporting two or more was 4–5 years.

**Table 2:** Estimates of total life expectancy according to the number of behavioural risk factors, by gender and cohort study England and Unites States 2002–2013.

	England (ELSA)		USA (HRS)	
	Years (95% CI)		Years (95% CI)	
	Men	Women	Men	Women
**Age 50**				
No behavioural risk factors	36.4 (35.8; 37.8)	40.1 (39.3; 41.4)	35.1 (34.4; 36.2)	38.9 (38.1; 39.5)
1 behavioural risk factor	32.5 (32.1; 33.2)	36.2 (35.6; 36.7)	30.7 (30.0; 31.6)	34.9 (34.4; 35.6)
2+ behavioural risk factors	28.4 (28.0; 29.3)	32.5 (31.7; 33.1)	27.0 (26.1; 28.3)	31.8 (31.0; 32.5)
**Age 60**				
No behavioural risk factors	27.3 (26.4; 28.7)	30.4 (29.6; 31.7)	26.0 (25.4; 26.7)	29.2 (28.6; 29.8)
1 behavioural risk factor	23.1 (22.5; 23.7)	26.9 (26.3; 27.4)	22.5 (22.0; 23.1)	25.5 (24.9; 25.9)
2+ behavioural risk factors	20.2 (19.9; 21.0)	24.2 (23.6; 24.7)	20.0 (19.5; 20.6)	23.0 (22.4; 23.6)
**Age 70**				
No behavioural risk factors	17.9 (16.9; 19.0)	21.2 (20.2; 22.4)	17.7 (17.2; 18.2)	20.1 (19.6; 20.8)
1 behavioural risk factor	14.9 (14.3; 15.4)	17.7 (17.2; 18.0)	14.7 (14.3; 15.1)	17.3 (16.9; 17.7)
2+ behavioural risk factors	12.9 (12.5; 13.3)	16.1 (15.6; 16.5)	13.6 (13.1; 14.1)	16.0 (15.5; 16.5)

Estimates from models with covariates age, sex, and wealth and interaction term between age and behavioural risk factors.

### Co-occurence of health behavioural risk factors and disability-free and chronic disease-free life expectancy

In Table 3 we report healthy life expectancy estimates at the age of 50, 60 and 70. Men in England with no behavioural risk factors could expect to live 33 additional years free of disability and, men in the USA 32 additional years; in women estimates were slightly higher (36 England and 34 USA). Life expectancy free from disability ranged between 23 and 26 years and, between 15 and 17 years at the ages of 60 and 70 respectively, in men and women in both countries with no behavioural risk factors. We found that, in both studies and at all ages, there was a clear gradient towards shorter disability-free life expectancy with increasing behavioural risk factors. For example, at the age of 50, compared to men with at least two behavioural risk factors, those with no behavioural risk factors could expect to live on average 10 years longer without disability in England and 11 years in the USA. The corresponding figures in women were 11 years in England and 10 years in the USA. At the age of 60 the difference between the two groups reduced to 9 and 7 years in men in England and the USA and, to 8 and 9 years in women in England and the USA respectively.

**Table 3:** Estimates of disability-free and chronic disease-free life expectancy according to the number of behavioural risk factors, by sex and cohort study England and Unites States 2002–2013.

	England (ELSA)			USA (HRS)	
	Years (95% CI)			Years (95% CI)	
	Men		Women	Men	Women
**Disability-free life expectancy**					
**Age 50**					
No behavioural risk factors	33.3 (32.5; 34.4)		35.7 (34.8; 36.8)	31.7 (30.7; 32.7)	33.5 (32.8; 34.2)
1 behavioural risk factor	28.8 (28.4; 29.4)		30.5 (29.7; 31.1)	26.6 (25.7; 27.7)	29.1 (28.4; 29.7)
2+ behavioural risk factors	23.4 (23.1; 24.4)		25.2 (24.4; 25.7)	21.1 (19.4; 22.8)	23.7 (22.8; 24.6)
**Age 60**					
No behavioural risk factors	24.4 (23.2; 25.7)		26.0 (25.0; 26.9)	22.6 (22.1; 23.3)	24.0 (23.5; 24.7)
1 behavioural risk factor	19.6 (19.0; 20.1)		21.8 (21.2; 22.3)	18.8 (18.3; 19.3)	19.5 (19.0; 19.9)
2+ behavioural risk factors	15.9 (15.5; 16.6)		17.8 (17.1; 18.3)	15.3 (14.7; 15.8)	15.3 (14.7; 15.8)
**Age 70**					
No behavioural risk factors	14.9 (13.9; 16.0)		17.0 (15.8; 18.0)	14.7 (14.2; 15.1)	15.1 (14.8; 15.9)
1 behavioural risk factor	11.7 (11.3; 12.2)		12.7 (11.9; 12.9)	11.2 (10.8; 11.6)	11.9 (11.5; 12.4)
2+ behavioural risk factors	9.2 (8.8; 9.7)		10.3 (9.7; 11.0)	9.5 (9.0; 10.0)	9.4 (8.8; 9.9)
**Chronic disease-free life expectancy**					
**Age 50**					
No behavioural risk factors		22.3 (20.5; 24.3)	26.1 (23.7; 27.7)	8.7 (6.7; 10.4)	7.9 (5.9; 9.5)
1 behavioural risk factor		19.4 (18.3; 20.4)	20.5 (18.5; 21.4)	5.4 (3.2; 6.9)	6.8 (4.7; 8.2)
2+ behavioural risk factors		13.3 (11.8; 14.5)	14.5 (12.8; 15.8)	4.7 (3.3; 6.1)	4.0 (2.7; 5.2)
**Age 60**					
No behavioural risk factors		14.3 (12.7; 17.8)	16.3 (14.4; 17.8)	4.7 (4.0; 5.4)	5.0 (4.1; 5.6)
1 behavioural risk factor		9.8 (8.6; 11.1)	11.4 (10.5; 12.9)	3.7 (3.2; 4.3)	3.6 (3.1; 4.1)
2+ behavioural risk factors		6.8 (5.7; 7.8)	7.3 (6.5; 8.4)	2.5 (2.0; 2.9)	1.9 (1.6; 2.3)
**Age 70**					
No behavioural risk factors		6.9 (5.5; 9.0)	10.1 (7.9; 11.1)	2.3 (1.8; 2.9)	2.5 (1.9; 3.1)
1 behavioural risk factor		4.4 (3.9; 5.4)	4.4 (3.7; 4.9)	1.6 (1.2; 1.9)	1.7 (1.4; 2.1)
2+ behavioural risk factors		3.3 (2.8; 4.0)	4.0 (3.1; 4.7)	1.0 (0.7; 1.4)	0.8 (0.6; 1.2)

Estimates from models with covariates age, sex, and wealth and interaction term between age and behavioural risk factors.

Chronic disease-free life expectancy at the ages of 50, 60 and 70 also decreased with increasing number of behavioural risk factors in both countries and genders. Chronic disease-free life expectancy was considerably shorter in the USA compared with England, even in the healthiest group. For example, men with no behavioural risk factors could expect to live an additional 9 years without chronic diseases in the USA and 22 in England.

### Individual health behavioural risk factors and disability-free and chronic disease-free life expectancy

**Figure 1:**
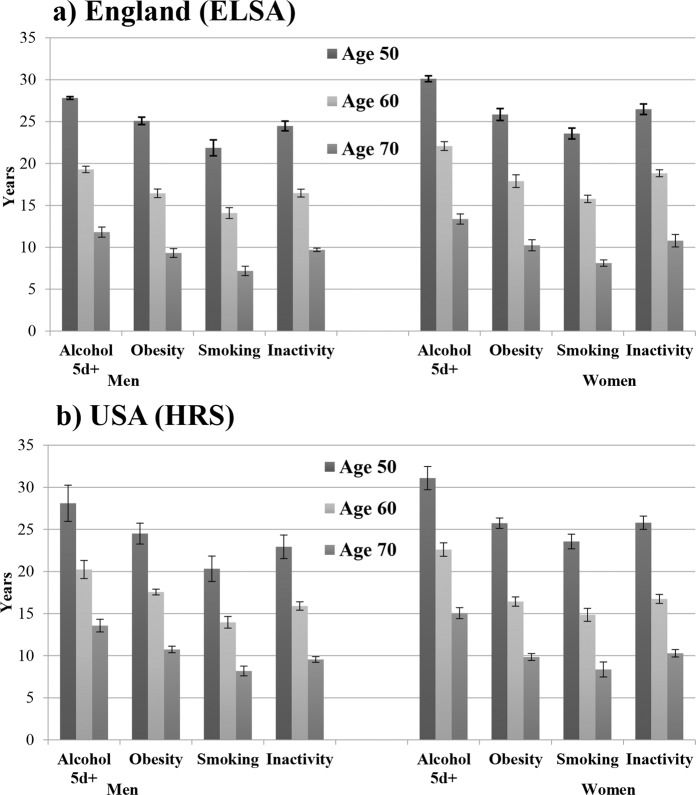
Disability-free life expectancy according to behavioural risk factors by sex, England and Unites States 2002–2013 panel (a) England (ELSA) panel (b) USA (HRS).

**Figure 2:**
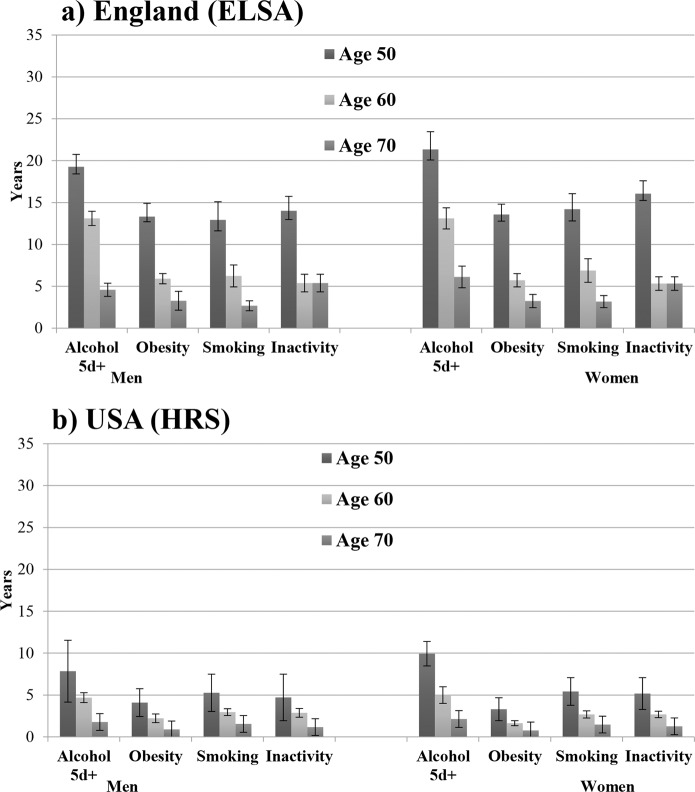
Chronic disease-free life expectancy according to behavioural risk factors by sex, England and Unites States 2002–2013, (**a**) England (ELSA) panel (b) USA (HRS).

In Figs. 1 and 2 we report the estimates of disability-free and chronic disease-free life expectancy, separately for each behavioural risk factors. Among men and women from England and the USA, smoking was associated with shortest estimated years of life free from disability at the ages of 50, 60 and 70 (Fig. 1).

The lowest number of years expected to live without chronic conditions, were observed among obese and physically inactive men and women from England (at all ages). In men and women from the USA, at the ages of 50 and 60, obesity was associated with the shortest estimated years of life without chronic conditions (Fig. 2).

### Sensitivity analysis

Given the substantially higher prevalence of arthritis reported by older Americans at baseline, we conducted a sensitivity analysis in which we excluded arthritis in the definition of chronic disease-free life expectancy (Supplementary Table S2). The estimated years expected to live without chronic conditions in English men and women were higher when excluding arthritis but, the pattern of inequalities by the number of behavioural risk factors were similar to those reported in Table 3. Men in the USA, with no behavioural risk factors, could expect to live an additional 15, 9 and 5 years without chronic conditions, at the ages of 50, 60 and 70 respectively (women 19, 13 and 8 years at the ages of 50, 60 and 70 respectively), compared to 9, 6 and 3 years, at the ages of 50, 60 and 70 respectively, among men with 2+ behavioural risk factors (women 11, 7 and 5 years at the ages of 50, 60 and 70 respectively). Although these estimates were higher than those reported when arthritis was included in the definition, the American disadvantage, compared to older English people, remained.

In order to investigate the possibility of reverse causation, we report estimates for people that were healthy at baseline (Supplementary Table S3). Differences in health expectancies by behavioural risk factors are similar to those reported in Table 3, suggesting that estimates are unlikely to be biased by health selection.

## Discussion

Using two large nationally representative studies of ageing, in the USA and England, we showed that clustered behavioural risk factors are associated with shorter life expectancy, as well as, shorter healthy life expectancy. At the ages of 50, 60 and 70, men and women with two or more of the behavioural risk factors (smoking, physical inactivity, obesity and alcohol consumption), could expect to live on average up to 12 fewer years than those with no risk factors. When estimating the remaining number of years spent without disability and without chronic conditions, we found in both studies a clear gradient towards shorter healthy life expectancy with increasing number of behavioural risk factors. We showed that, of the remaining years of life at the age of 50, up to 11 years spent without disability were lost in people with two or more of the behavioural risk factors, compared to people with none, and up to 12 for chronic disease-free life expectancy. Patterns observed were comparable in the two studies for disability-free life expectancy, but chronic disease-free life expectancy was substantially shorter in the USA than English samples. However, the impact of cumulative behavioural risk factors was similar.

Furthermore, of the individual behavioural risk factors, current smoking and obesity were associated with shortest estimates of disability and chronic disease-free life expectancy in England and the USA.

Our results are in accordance with studies among older adults aged <75^8,9,11,12,14^, but by exploring both disability-free and chronic disease-free healthy life expectancy among older individuals aged 50+ from the USA and England we have extended previous work. Given that many people are expected to live beyond the age of 75, understanding how many of these years will be spent in good health and which factors might be contributing to longer and healthier lives is crucial for policy makers.

In both studies, chronic disease-free life expectancy was shorter than disability-free life expectancy. This was expected, as the baseline prevalence of chronic conditions was higher than the prevalence of disability and, recovery was not allowed in the computation of chronic disease-free life expectancy; whereas, the transition from disability to non-disability was possible. Our results also revealed that chronic disease-free life expectancy was much shorter in the USA study compared to the English study. A health disadvantage of older people in the USA compared to England in terms of several chronic conditions^23,25^ has been previously reported and, is not due to biases in self-reported disease because objective health measures, such as biological markers, showed the same pattern^25^. Older Americans in this study reported considerably higher rates of arthritis, and other chronic conditions than comparable people from England (Supplementary Table S1). In Supplementary Table S2 we report estimates of chronic disease-free life expectancy computed excluding arthritis. We found that, although estimates were higher for older Americans compared to estimates which included arthritis in the definition, the disadvantage compared to older English people remained and ranged between 5 and 8 years. One possible explanation for shorter chronic disease-free life expectancy might relate to differences in welfare state systems between England and the USA. A wide range of social protection schemes is available in England, including unemployment compensation, sick pay, housing policies, and social retirement benefits. The generosity and coverage of social protection have significant impacts on health, result in better psychosocial health and reduced stress. Older Americans might be exposed to more psychosocial distress and, might be at higher risk of reporting chronic conditions and early mortality^22,26^.

Both studies showed an apparent advantage of those who consumed alcohol for 5 days a week or more, compared to those who consumed it less frequently. It is possible that those drinking less frequently had to reduce alcohol intake due to ill health. We acknowledge that, due to the crude information about frequency of alcohol consumption collected in the two surveys, we have not been able to distinguish between moderate and heavy drinkers.

The results of our study should be considered in the light of some limitations. The definition of chronic health conditions used in this study included diagnoses that occurred also before the age 50, however, we were not able to estimate the impact of health behaviours on loss of years lived without chronic disease occurring before the age of 50. Although the two studies were designed to be comparable and we made use of harmonized data from the Gateway to Global Aging Data, there is still potential for residual inconsistencies in the definition of variables. For example, in ELSA frequency of alcohol consumption was ascertained in the 7 days prior the interview and, in HRS in the three months prior the interview. Body weight and height were self-reported in HRS and objectively measured by a nurse in England (ELSA). As a consequence, the prevalence of obese people in HRS might be underestimated. In our study we could only consider frequency of vigorous physical activity (3+ days a week in the USA (HRS) and 2+ days a week in England (ELSA)), because at the baseline year of our study, HRS only provided this information, whereas in ELSA more detailed information of physical activity intensity and frequency were available. This resulted in a high prevalence of older people being classified as physically inactive, whereas a measure based on light and moderate physical exercise would have been preferable. Another limitation of our study is the use of self-reported chronic conditions. Medical records for chronic conditions would have been preferable. Results from previous studies showed that self-reports of chronic conditions and cancer were reasonably accurate^27,28^. Furthermore, in both surveys, the wording of the questions on chronic conditions has been formulated to reduce subjectivity (“Has a doctor ever told you that you have …”)^22^. Lastly, our estimates of chronic disease-free life expectancy and disability-free life expectancy might be overestimated if respondents in our samples are a selected group of healthier people and mortality rates do not match those observed nationally in the US and England. To further explore these possibilities, we reported estimates for people that were healthy at baseline, the results were similar suggesting that, estimates are unlikely to be biased by health selection. Furthermore, we compared estimates of total life expectancy obtained from our data with national life tables and, found similar results for England and slightly higher estimates for the US compared to life tables^22,29,30^.

To conclude, we have shown that the co-occurrence of unhealthy behavioural risk factors reduces considerably the number of remaining years of life expected to live without disability and without chronic conditions. The similarity of the associations in these two countries with different public health and health care systems and different cultural expectations highlights the robustness of effect. Reducing smoking, obesity and increasing physical activity among older people could potentially lead not only to longer lives but also healthier lives.

### Ethical approval

Ethical approval for HRS was obtained by the relevant committees at the University of Michigan and the US National Institute on Aging, the primary sponsor of HRS. For ELSA ethical approval and experimental protocols were granted by the Multi-centre Research and Ethics Committee (MREC). Respondents in HRS and ELSA gave their informed consent to participate in the study and for data linkage. The authors confirm that all research was performed in accordance with relevant guidelines/regulations.

## Supplementary information

Supplementary Information.

## Data Availability

The data used in this work can be obtained free upon registration at the https://g2aging.org/; codes to compute healthy life expectancy can be downloaded at http://sites.utexas.edu/space/.
