# Supplementary material for: Behavioural risk factors and healthy life expectancy: evidence from two longitudinal studies of ageing in England and the US
Source: Sci Rep. 2020 Apr 24;10:6955. doi: 10.1038/s41598-020-63843-6 (PMC7181761; doi:10.1038/s41598-020-63843-6)
Supplement: Supplementary file 1 — Supplementary Information. [file 41598_2020_63843_MOESM1_ESM.doc]

**Title: Behavioural risk factors and healthy life expectancy: evidence from two longitudinal studies of ageing in England and the US**

**Paola Zaninotto, Jenny Head, Andrew Steptoe**

**Supplementary Materials**

**1. Harmonization of the measures used**

For the analyses of this paper we used ELSA (for England) and HRS (for the USA) harmonized data available from The Gateway to Global Aging Data (g2aging.org). The Gateway to Global Aging Data is a project sponsored by the National Institute on Aging, to facilitate cross-national comparative studies on aging using a family of health and retirement studies around the world. Harmonized datasets are developed as a single file which includes all longitudinal waves of the study of interest. Harmonized variables use a consistent and intuitive variable naming convention across surveys to identify directly comparable measures between studies. Each Harmonized data file has its own codebook which carefully explains how each harmonized variable was created and highlights any differences between the same variable in different studies. The HRS harmonized data documentation^1^ is available at

<http://hrsonline.isr.umich.edu/modules/meta/rand/randhrsp/randhrs_P.pdf>

And the ELSA harmonized data documentation^2^ is available at

<http://doc.ukdataservice.ac.uk/doc/5050/mrdoc/pdf/5050_harmonized_elsa_e.pdf>

***Chronic conditions***

In both studies the respondents are asked whether the doctors ever told them they have chronic medical conditions. From each harmonized data we selected the following chronic conditions at each wave: coronary heart disease, stroke, chronic lung disease, cancer (excluding minor skin cancer), diabetes and arthritis (or rheumatism). More information on these variables can be found on page 502 of reference 1 and page 165 of reference 2.

***Activities of daily living and Instrumental activities of daily living***

In both studies difficulties with activities of daily living (bathing, eating, dressing, using the toilet, walking across a room, and getting in or out of bed) and instrumental activities of daily living (using a telephone, taking medication, handling money, shopping, preparing meals) are asked to respondents at each wave and include the same response categories. More information on these variables can be found on page 292 of reference 1 and page 165 of reference 2.

***Behavioural risk factors***

Body Mass Index is computed using body weight and height which in HRS are self-reported, while in ELSA study nurses measured weight and height in the participants’ homes at alternative waves. Questions about smoking status are fully comparable in the two studies (whether the respondent ever smoked cigarettes and whether smokes cigarettes nowadays). In ELSA frequency of alcohol consumption indicates the number of days the respondent reported having an alcoholic drink in the last seven days, while in HRS in the last three months. Participation in vigorous physical activity was asked in both HRS and ELSA and recorded as the number of days participants took part in sports or activities that are vigorous. The main difference between the two studies is that HRS records only whether the respondent was physically active for 3 or more days a week and ELSA for 2 or more.

***Socioeconomic indicators***

HRS and ELSA both contain high-quality wealth modules using a comprehensive and detailed set of questions on the components of wealth^3^. Total family net non-pension wealth in both studies is defined as the sum of financial wealth, physical wealth (such as business wealth, land, or jewels), and housing wealth (primary and secondary residential housing wealth), minus all debts. For more details the readers can refer to section C of the HRS harmonized documentation file (page 854 of reference 1) and Section E of the ELSA harmonized documentation file (page 297 of reference 2). The continuous variable of wealth within each study was recoded into equally sized tertiles.

**2 Computation of healthy life expectancy**

The computation of health expectancy using the Sullivan method is usually applied to cross-sectional data, and requires life tables and information on age specific proportions of the population in healthy or unhealthy stages. These proportions are prevalence measures of the actual and current health status of a real population and are used to divide years lived in the life table population. With panel data reliable estimates of life table inputs cannot be obtained.

The multi-state life table (MSLT) model has been developed to analyse stochastic processes that involve multiple and recurrent events (typical of longitudinal data), in order to estimate expected duration in various states. The MSLT method uses a set of transition probabilities from

healthy, unhealthy and to death estimated using longitudinal data. We defined the following three

states: healthy, unhealthy and dead. For disability-free life expectancy there were four possible

transitions between the states, namely: healthy to unhealthy (onset), unhealthy to healthy

(recovery), healthy to dead, unhealthy to dead. For chronic disease-free life expectancy there were

only three possible transitions as, by definition, recovery was not possible. The advantages of

multistate life table methods are: it is based on incidence measures representing current health

transitions; it allows movement in both directions between all surviving health states; it allows death

rates to differ by health state so it takes into account the different mortality profiles by health status.

The estimation of transition schedules is very important and can be done using logistic regression,

multinomial logistic regression, or proportional hazards regression.

We used the Stochastic Population Analysis for Complex Events (SPACE)^4^ program in SAS 9.2 to

estimate MSLT functions. There are two main components to this program: the data component,

which prepares the input datasets, and the statistical component in which transition probabilities and

the multistate life table functions and their variances are estimated. Specifically, during the statistical

component age-specific transition probabilities for all possible transitions are estimated from the data

using multinomial logistic regression with age, sex, wealth tertiles, behavioural risk factors and the interaction term between age and behavioural risk factors. Health expectancies for ages 50+ are then calculated based on these estimated transition probabilities using a stochastic (micro-simulation) approach. By using microsimulation it is possible to simulate the life paths of the members of the population in order to derive several summary statistics of the population dynamics. For each study separately, the program generated individual trajectories for a simulated cohort of 100,000 persons with distributions of covariates at the starting point based on the observed study-specific prevalence by five year age group and sex. Analyses were run for combined behavioural risk factors and for each of the four behavioural risk factors separately. Variability for these multistate life table estimates (variances, standard errors and corresponding 95% confidence intervals) were computed using a bootstrap method with 500 replicates for the whole analysis process (multinomial analysis and simulation steps).

More information can be found at <http://www.cdc.gov/nchs/data_access/space.htm>.

The SPACE manual is available at <ftp://ftp.cdc.gov/pub/Health_Statistics/NCHS/Software/space/SPACE_manual.pdf>

The annotated codes can be found at

<ftp://ftp.cdc.gov/pub/Health_Statistics/NCHS/Software/space/sas/MSLT_RAD2COV_S.pdf>

<ftp://ftp.cdc.gov/pub/Health_Statistics/NCHS/Software/space/sas/MSLT_SIMxCOV_S.pdf>

ftp://ftp.cdc.gov/pub/Health_Statistics/NCHS/Software/space/sas/MSLT_SIMxCOV_M_DX.pdf

<ftp://ftp.cdc.gov/pub/Health_Statistics/NCHS/Software/space/sas/SMPEM_SIMDUR.pdf>

<ftp://ftp.cdc.gov/pub/Health_Statistics/NCHS/Software/space/sas/SMPEM_SIMxCOV_S.pdf>

**Table S1 Baseline prevalence of chronic diseases according by sex, England and Unites States 2002. All table figures are column percentages.**

|  | **England**  **(ELSA)** | | **USA**  **(HRS)** | |
| --- | --- | --- | --- | --- |
| **Condition** | **Men**  (n**=**4,718) | **Women**  (n**=**5,620) | **Men**  (n**=**7,308) | **Women**  (n**=**10,043) |
| Coronary heart disease | 15.5 | 10.3 | 25.6 | 19.7 |
| Stroke | 4.3 | 3.4 | 7.9 | 7.4 |
| Lung disease | 6.7 | 6.1 | 8.0 | 8.1 |
| Cancer | 5.0 | 7.1 | 12.8 | 13.0 |
| Diabetes | 8.5 | 5.7 | 17.5 | 14.2 |
| Arthritis | 24.6 | 37.3 | 47.5 | 61.1 |
| Chronic health conditions excluding arthritis | 29.4 | 24.4 | 56.2 | 48.3 |

Percentages are estimated using sampling weights

**Table S2 Chronic disease-free life expectancy (excluding arthritis) according to the number of behavioural risk factors by sex, England and Unites States,** 2002-2013

|  | | **England (ELSA)** | | | **USA (HRS)** | |
| --- | --- | --- | --- | --- | --- | --- |
|  |  | **Years (95% CI)** | | | **Years (95% CI)** | |
|  | | **Men** | | **Women** | **Men** | **Women** |
|  |  |  |  |  |  |  |
| **Chronic disease-free life expectancy** | | | |  |  |  |
| **Age 50** |  | |  |  |  |  |
| No behavioural risk factors | | 21.4 (19.8; 22.1) | | 26.3 (24.3; 27.1) | 15.0 (13.2; 14.8) | 18.8 (17.0; 18.9) |
| 1 behavioural risk factor | | 20.2 (19.2; 20.9) | | 23.2 (21.1; 24.3) | 12.0 (10.4; 12.4) | 15.8 (14.6; 15.2) |
| 2+ behavioural risk factors | | 13.9 (13.0; 14.7) | | 17.3 (15.8; 18.1) | 9.2 (7.4; 9.2) | 11.4 (10.5; 10.8) |
| **Age 60** |  | |  |  |  |  |
| No behavioural risk factors | | 15.8 (15.3; 17.5) | | 19.7 (19.4; 20.0) | 9.3 (8.9; 9.1) | 13.4 (12.5; 12.8) |
| 1 behavioural risk factor | | 12.6 (12.0; 14.0) | | 16.7 (16.0; 17.3) | 7.4 (7.4; 7.8) | 10.4 (10.0; 10.3) |
| 2+ behavioural risk factors | | 9.1 (7.8; 10.1) | | 12.2 (12.0; 13.5) | 5.5 (5.8; 6.4) | 7.3 (7.0; 7.2) |
| **Age 70** |  | |  |  |  |  |
| No behavioural risk factors | | 9.0 (8.8; 10.1) | | 13.9 (13.4; 14.6) | 4.9 (4.4; 5.0) | 8.3 (8.2; 8.4) |
| 1 behavioural risk factor | | 6.2 (5.7; 6.4) | | 8.5 (38.1; 8.9) | 3.3 (2.9; 3.4) | 6.1 (5.8; 6.2) |
| 2+ behavioural risk factors | | 4.8 (4.2; 4.9) | | 7.3 (7.1; 8.0) | 2.6 (2.5; 2.9) | 4.5 (4.2; 4.6) |

Estimates from models with covariates age, sex, and wealth and interaction term between age and behavioural risk factors

**Table S3 Estimates of disability-free and chronic disease-free life expectancy according to the number of behavioural risk factors in healthy people at baseline, by sex and cohort study England and Unites States 2002-2013**

|  | | **England (ELSA)** | | | **USA (HRS)** | |
| --- | --- | --- | --- | --- | --- | --- |
|  |  | **Years (95% CI)** | | | **Years (95% CI)** | |
|  | | **Men** | | **Women** | **Men** | **Women** |
|  |  |  |  |  |  |  |
| **Disability-free life expectancy** | |  | |  |  |  |
| **Age 50** |  |  | |  |  |  |
| No behavioural risk factors | | 33.4 (32.5; 34.5) | | 35.8 (34.9; 36.9) | 32.0 (31.0; 32.8) | 33.7 (33.0; 34.4) |
| 1 behavioural risk factor | | 29.0 (28.7; 29.6) | | 30.8 (29.9; 31.2) | 27.2 (26.5; 28.1) | 29.3 (28.7; 30.0) |
| 2+ behavioural risk factors | | 24.2 (23.8; 24.9) | | 25.8 (25.0; 26.3) | 23.2 (22.0; 24.2) | 24.5 (23.7; 25.2) |
| **Age 60** |  |  | |  |  |  |
| No behavioural risk factors | | 24.6 (23.4; 25.8) | | 26.2 (23.4; 25.8) | 22.8 (22.3; 23.5) | 24.2 (23.7; 24.9) |
| 1 behavioural risk factor | | 20.1 (19.5; 20.6) | | 22.0 (19.5; 20.6) | 19.2 (18.8; 19.7) | 20.2 (19.8; 20.5) |
| 2+ behavioural risk factors | | 16.8 (16.5; 17.5) | | 18.6 (18.0; 18.9) | 16.2 (15.7; 16.7) | 16.5 (16.0; 17.0) |
| **Age 70** |  |  | |  |  |  |
| No behavioural risk factors | | 15.1 (14.3; 16.3) | | 17.3 (16.2; 18.3) | 15.0 (14.5; 15.4) | 15.4 (15.0; 16.1) |
| 1 behavioural risk factor | | 12.4 (11.9; 12.8) | | 13.6 (13.0; 13.9) | 12.0 (11.7; 12.4) | 12.8 (12.5; 13.2) |
| 2+ behavioural risk factors | | 10.1 (9.9; 10.5) | | 11.7 (11.1; 12.0) | 10.5 (10.0; 10.9) | 10.8 (10.3; 11.3) |
| **Chronic disease-free life expectancy** | | | |  |  |  |
| **Age 50** |  | |  |  |  |  |
| No behavioural risk factors | | | 26.6 (24.7; 28.2) | 31.0 (27.7; 31.7) | 11.7 (10.7; 13.0) | 12.6 (11.6; 13.6) |
| 1 behavioural risk factor | | | 23.0 (22.2; 24.6) | 26.2 (24.8; 27.2) | 10.8 (9.7; 11.6) | 11.6 (10.6; 12.6) |
| 2+ behavioural risk factors | | | 17.4 (16.5; 18.7) | 20.0 (18.8; 20.8) | 8.4 (7.5; 9.3) | 9.0 (7.9; 10.0) |
| **Age 60** |  | |  |  |  |  |
| No behavioural risk factors | | | 21.4 (19.7; 23.3) | 24.0 (21.8; 25.3) | 10.0 (9.3; 10.8) | 10.9 (10.3; 11.5) |
| 1 behavioural risk factor | | | 16.7 (16.3; 17.9) | 19.7 (18.7; 20.8) | 9.2 (8.6; 9.7) | 10.0 (9.4; 10.5) |
| 2+ behavioural risk factors | | | 13.9 (13.3; 14.8) | 16.4 (15.8; 17.3) | 7.4 (6.8; 8.0) | 8.0 (7.4; 8.7) |
| **Age 70** |  | |  |  |  |  |
| No behavioural risk factors | | | 15.0 (13.5; 16.1) | 17.7 (15.8; 18.8) | 8.1 (7.6; 8.9) | 8.9 (8.3; 9.6) |
| 1 behavioural risk factor | | | 10.9 (10.6; 11.9) | 12.6 (12.0; 13.0) | 7.4 (6.9; 7.9) | 8.1 (7.7; 8.7) |
| 2+ behavioural risk factors | | | 9.5 (9.0; 10.2) | 11.9 (11.0; 12.4) | 6.4 (5.7; 7.0) | 6.8 (6.2; 7.8) |

Estimates from models with covariates age, sex, and wealth and interaction term between age and behavioural risk factors

**References**

1. Delia Bugliari NC, Chris Chan, Orla Hayden, Michael Hurd, Regan Main, Joshua Mallett, Colleen McCullough, Erik Meijer, Michael Moldoff, Philip Pantoja, Susann Rohwedder, Patricia St.Clair. RAND HRS Data Documentation, Version P. 2016.

2. Drystan Phillips Y-CL, Jenny Wilkens, Sandy Chien, Michael Moldoff, Jinkook Lee, Gema Zamarro. Harmonized ELSA Documentation. 2016.

3. Banks J MA, Smith JP. Attrition and health in ageing studies: evidence from ELSA and HRS. *Longitudinal and Life Course Studies* 2011;2(2):101-26. <https://doi.org/10.14301/llcs.v2i2.115>

4. Cai L, Hayward MD, Saito Y, et al. Estimation of multi-state life table functions and their variability from complex survey data using the SPACE Program. *Demogr Res* 2010;22(6):129-58. <https://doi.org/10.4054/DemRes.2010.22.6>
